# Peripheral Color Demo

**DOI:** 10.1177/2041669515613671

**Published:** 2015-11-06

**Authors:** Christopher W Tyler

**Affiliations:** Division of Optometry and Vision Science, City University, London, UK; Smith-Kettlewell Eye Research Institute, San Francisco, Ca, USA

**Keywords:** Periphery, color vision, eccentricity, cone distribution, fovea

## Abstract

A set of structured demonstrations of the vividness of peripheral color vision is provided by arrays of multicolored disks scaled with eccentricity. These demonstrations are designed to correct the widespread misconception that peripheral color vision is weak or nonexistent.

There is a widespread misconception even among vision scientists, and hence the population in general, that the high cone density in the fovea implies that color vision is restricted to the central vision, and conversely that the high density of rods in the periphery implies a lack of color vision in the periphery. For example, the Wikipedia article on peripheral vision says

Peripheral vision is weak in humans, especially at distinguishing colour and shape … rod cells are unable to distinguish colour and are predominant at the periphery, while cone cells are concentrated mostly in the centre of the retina, the fovea. (https://en.wikipedia.org/wiki/Peripheral_vision)

In fact, however, both historical (Østerberg, 1935) and more recent (Curcio, Sloan, Packer, Hendrickson, Kalina, 1987; Song, Chui, Zhong, Elsner, & Burns, 2011) measurements of photoreceptor densities indicate otherwise. Despite the high concentration of cones in the fovea, even the central 5° of the retina contains only about 50,000 cones (1% of the total), while the remainder of the total population of about 5 million cones is distributed throughout the peripheral retina with an average density of about 5,000 cones/mm^2^ (beyond about 10° eccentricity). Since the cone inner segments act as their light-catching apertures, and since their diameter is about 10 mm in peripheral retina (Jonas, Schneider & Naumann, 1992), this density implies that the light-catching area of the cones is about 0.3 mm^2^ per mm^2^ of peripheral retina, while the rod light-catching area accounts for most of the rest. Thus, about one third of the peripheral retina should be considered to support color vision (Williams, 1991).

The mapping from retina to cortex can be approximated as a linear scaling from the fovea to the periphery, particularly for the cortical mappings of V2 and V3 (Schira, Tyler, Breakspear, & Spehar, 2009). To project from the retina to equal regions of early visual cortex, therefore, the stimuli should be scaled in proportion to eccentricity, and studies of peripheral color processing should use such scaling in order to assess the cortical capabilities of color processing. Indeed, with suitable areal scaling, color discrimination can be equated at all eccentricities This is not the place for an extensive review, but it should be noted that many studies of peripheral processing have used constant stimulus size and report progressive declines in hue discrimination (Nagy & Wolf, 1993; McKeefry, Murray & Parry, 2007; Mullen, 1991), chromatic saturation (Stabell & Stabell, 1982; Abramov, Gordon & Chan, 1992, McKeefry, Murray & Parry, 2007; Volbrecht & Nerger, 2012), and conspicuity (Gunther, 2014). Those studies using appropriate size scaling of the stimuli generally find approximate invariance of the processing properties as a function of eccentricity (Noorlander, Koenderink, den Ouden, & Edens, 1983; Rovamo & Iivanainen, 1991; Abramov, Gordon & Chan, 1991, 1992; Sakurai & Mullen, 2006). Where significant effects are reported, one may question the precise choice of scaling factor. Tyler (1987a), for example, proposed that the appropriate scaling factor should be based on stimulating the same number of cones at each eccentricity, whereas most scaling studies attempt to equate the number of ganglion cells stimulated or the cortical magnification factor per se (although some such studies do not apply this logic to the central foveal stimuli).

These properties are indicated by the following demonstration images, which should be viewed at a distance so as to make the width of the central disk measure about one twelfth of the viewing distance (e.g., 1 inch diameter at a 12 inch viewing distance). This ratio corresponds to about the most liberal definition of the foveal region (5° diameter). Figure 1 shows an array of multicolored “balloons” within this foveal region scaled to stimulate about 1 cm^2^ of visual cortex at each eccentricity. In Figure 2, the same form of array is scaled up to project to the periphery beyond the foveal limit (2.5° eccentricity) into the periphery. If periphery color vision had weaker color vision, the colors when viewing Figure 2 should appear desaturated relative to those in Figure 1, but inspection verifies that they do not. (To reach the farther periphery, the viewing distance should be reduced to 5 inches with the 1 inch central disk region, when the outer rim will reach out to 45° eccentricity with central fixation.) Figure 2 also includes an overlay of a horizontal line of unscaled disks typical of unscaled studies of peripheral color vision, to allow comparison of its degradation with eccentricity under these conditions.

**Figure 1.**
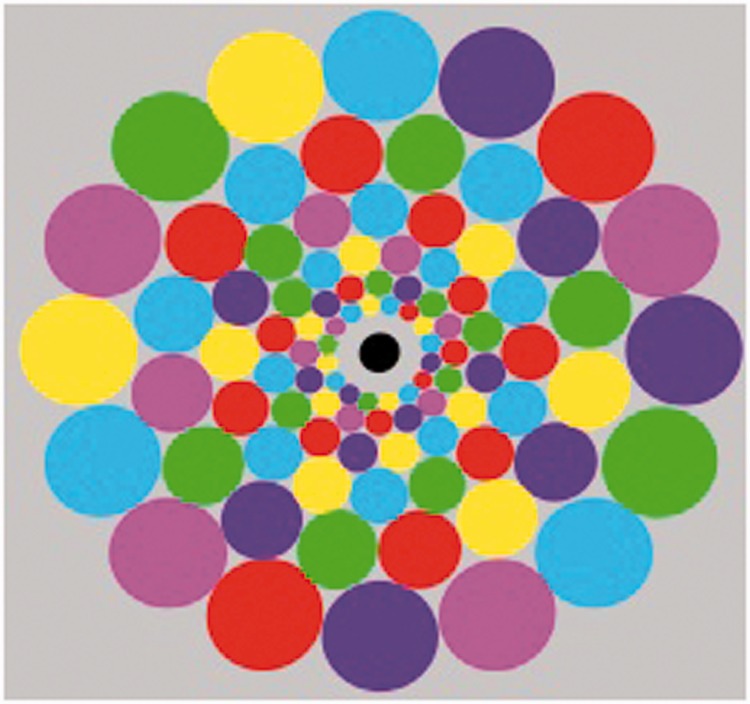
Foveal color vividness demo designed to be viewed at two viewing distances. At a viewing distance of 12× the circular array width it spans the 5° fovea where the variegated color disks show the vividness of foveal color perception. At 60× the circular array width, it spans the 1° foveola, illustrating fine-resolution color processing.

**Figure 2.**
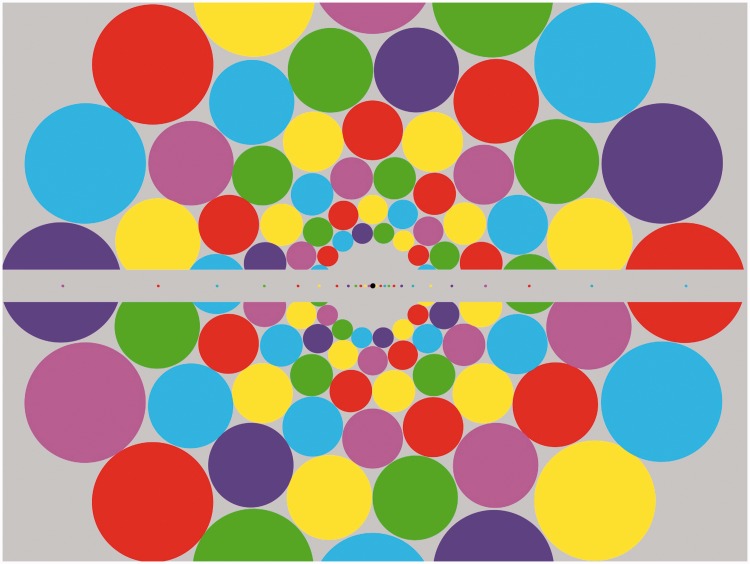
Peripheral color vividness demo designed for two viewing distances when fixating steadily at the centre. At 12 – the central gray disk width, it spans the periphery from 2.5° to 20°. At the close-up distance of 3× the central gray disk width, it spans from about 10° to 50° eccentricity, illustrating the vividness of peripheral color processing. The central gray bar contains elements that are unscaled for eccentricity, to illustrate the perceptual fall off in peripheral color perception.

Given that the natural units of cortical processing characterized by the concept of the “hypercolumn” are of the order of 2 mm wide in human visual cortex, the disks in Figures 1 and 2 should each stimulate about 25 such units. To check whether color processing is similarly uniform at a grain of about 1 hypercolumn, the sizes of each disk are reduced by a factor of five for the peripheral version in Figure 3. It can be seen that color is again visible out to the edge of the image without noticeable desaturation under these reduced stimulation conditions, so integration across multiple hypercolumnar units is not required to support peripheral color processing.

**Figure 3.**
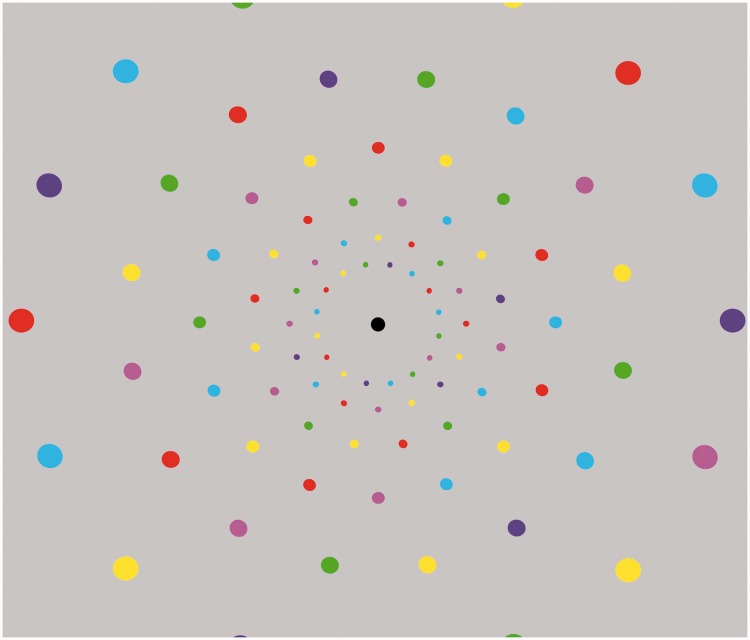
Peripheral variegated color disks at 5× reduced scale show that peripheral color remains as vivid even close to the spatial summation limit.

If anything, inspection of the figures shows the color perception is more vivid in the periphery, as might be expected from the fact that the cone density decreases at a slower rate than linear reciprocity with eccentricity (Curcio et al., 1987). In fact, the cone density scales with approximately the −2/3 power of eccentricity out to 20° (Tyler, 1987b). Thus, the linear scaling of the disk sizes should result in the stimulation of about five times [(10^−2/3^/10^−1^)^2^] more cones by 20° than 2° in areal terms, giving scope for cortical processing to account for the extra vividness that is perceptually observed.
